# Maternal Factor Dppa3 Activates 2C-Like Genes and Depresses DNA Methylation in Mouse Embryonic Stem Cells

**DOI:** 10.3389/fcell.2022.882671

**Published:** 2022-06-03

**Authors:** Chuanyu Zhang, Hang Wen, Siying Liu, Enze Fu, Lu Yu, Shang Chen, Qingsheng Han, Zongjin Li, Na Liu

**Affiliations:** ^1^ School of Medicine, Nankai University, Tianjin, China; ^2^ Key Laboratory of Bioactive Materials, Ministry of Education, College of Life Sciences Nankai University, Tianjin, China

**Keywords:** Dppa3, DNA methylation, 2CLCs, Embryonic stem cells, Zscan4

## Abstract

Mouse embryonic stem cells (ESCs) contain a rare cell population of “two-cell embryonic like” cells (2CLCs) that display similar features to those found in the two-cell (2C) embryo and thus represent an *in vitro* model for studying the progress of zygotic genome activation (ZGA). However, the positive regulator determinants of the 2CLCs’ conversion and ZGA have not been completely elucidated. Here, we identify a new regulator promoting 2CLCs and ZGA transcripts. Through a combination of overexpression (OE), knockdown (KD), together with transcriptional analysis and methylome analysis, we find that Dppa3 regulates the 2CLC-associated transcripts, DNA methylation, and 2CLC population in ESCs. The differentially methylated regions (DMRs) analysis identified 6,920 (98.2%) hypomethylated, whilst only 129 (1.8%) hypermethylated, regions in *Dppa3* OE ESCs, suggesting that Dppa3 facilitates 2CLCs reprogramming. The conversion to 2CLCs by overexpression of *Dppa3* is also associated with DNA damage response. *Dppa3* knockdown manifest impairs transition into the 2C-like state. Global DNA methylome and chromatin state analysis of *Dppa3* OE ESCs reveal that *Dppa3* facilitates the chromatin configuration to 2CLCs reversion. Our finding for the first time elucidates a novel role of Dppa3 in mediating the 2CLC conversion, and suggests that Dppa3 is a new regulator for ZGA progress.

## Introduction

Zygotic genome activation (ZGA) refers to the activation and transcription of paternal and maternal genomes after fertilization, which is a key event in development (Rossant and Tam, 2017). The maternal to zygotic transition (MZT) marks the time the embryonic genome is activated and acquires control of development. The major ZGA occurs predominantly at the two-cell (2C) stage of mouse embryo, where zinc finger and scan domains containing four clusters (Zscan4) is thought to be specifically expressed during preimplantation ZGA (Li et al., 2013; Lee et al., 2014; Jukam et al., 2017). Zscan4 family comprises nine very closely related gene paralogues located on mouse chromosome 7. Falco *et al.* found *Zscan4d* as a gene upregulated during zygotic genome activation in mouse two-cell stage embryos (Falco et al., 2007; Storm et al., 2009). In mice, only zygote and 2C-stage embryo are truly totipotent that can give rise to embryos and all their supporting extra-embryonic tissues.

Mouse embryonic stem cells (ESCs) are prototypical pluripotent cells, which are derived from the inner cell mass (ICM) of blastocysts (Evans and Kaufman, 1981; Martin, 1981). Embryonic stem cells are an *in vitro* model for studying the early embryos around the implantation stage. ESCs are heterogeneous and contain subpopulations with different properties (Hayashi et al., 2008; Toyooka et al., 2008; Macfarlan et al., 2012). One of these subpopulations is “2-cell embryonic like cells (2CLCs)” (Zalzman et al., 2010; Macfarlan et al., 2012), which shared features with the two-cell embryo, contributing to both embryonic and extra-embryonic tissues (trophoblast) (Zalzman et al., 2010; Macfarlan et al., 2012). 2CLCs occur spontaneously in ESCs, but the probability of occurrence is very low, only 0.1–0.4% (Hu et al., 2020). 2CLCs express high levels of ZGA transcripts, including mouse endogenous retroviruses (ERV)-L (*MERVL*) family retroviruses and Zscan4 (Macfarlan et al., 2012; Eckersley-Maslin et al., 2016). 2CLCs model essential aspects of the two-cell stage embryos, which can be used to investigate the molecular regulator for ZGA (Eckersley-Maslin et al., 2018). Using this model, recently, several positive regulators (including Dux (De Iaco et al., 2017; Hendrickson et al., 2017; Yang et al., 2020), Dppa2/4 (De Iaco et al., 2017; Eckersley-Maslin et al., 2019; Yan et al., 2019), and Zscan4 (Zalzman et al., 2010)), and negative regulators (Ythdc1 (Liu et al., 2021), CTCF (Olbrich et al., 2021), Lin28 (Sun et al., 2021), and Kap1/Trim28 (Maksakova et al., 2013)) of ZGA were reported.

Maternally inherited factors play important roles in the zygotic genome activation during early development (Li et al., 2010; Ancelin et al., 2016; Wasson et al., 2016). Developmental pluripotency-associated 3 (*Dppa3,* also known as *Stella* or *PGC7*) is a maternally inherited factor (Funaki et al., 2014; Huang et al., 2017). Zygotes lacking maternal Dppa3 actively fail to reach the blastocyst stage (Payer et al., 2003; Bortvin et al., 2004), demonstrating that Dppa3 plays a role during the cleavage stages of pre-implantation development. Embryonic gene activation from the paternal *Dppa3* allele at the two-cell stage does not rescue the abnormalities resulting from maternal deletion, indicating that a crucial function of Dppa3 must occur after fertilization but before the two-cell stage (Nakamura et al., 2007). *Dppa3* is also expressed in naïve embryonic stem cells, but is downregulated upon exit from pluripotency (Hayashi et al., 2008; Sang et al., 2019; Zhao et al., 2019). Although some functions of Dppa3 in DNA methylation have been discovered, however, little is known about the functions and mechanisms of Dppa3 on 2C-like state and ZGA.

Dux and Dppa2/4, key factors enhancing ZGA, were successively reported as non-essential for ZGA activation *in vivo*, indicating that there are other key regulators that have not yet been revealed (De Iaco et al., 2017; Chen and Zhang, 2019; Guo et al., 2019; De Iaco et al., 2020; Chen et al., 2021; Kubinyecz et al., 2021). Because Dppa3 is expressed initially as an oocyte factor and because maternal factors are so important for ZGA, we tested whether Dppa3 plays a role in 2CLCs conversion and ZGA. Here, we use the 2CLCs system to identify Dppa3 as a new regulator for 2C-like gene activation. An additional DNA methylation analysis further indicates a role for Dppa3 in establishing hypomethylated chromatin to allow correct gene regulation. These results reveal the key developmental roles of Dppa3 in the regulation of 2CLCs conversion in ESCs.

## Results

### Dppa3 Promotes a 2C-Like State and ZGA Specific Gene Expression in ESCs

**FIGURE 1:**
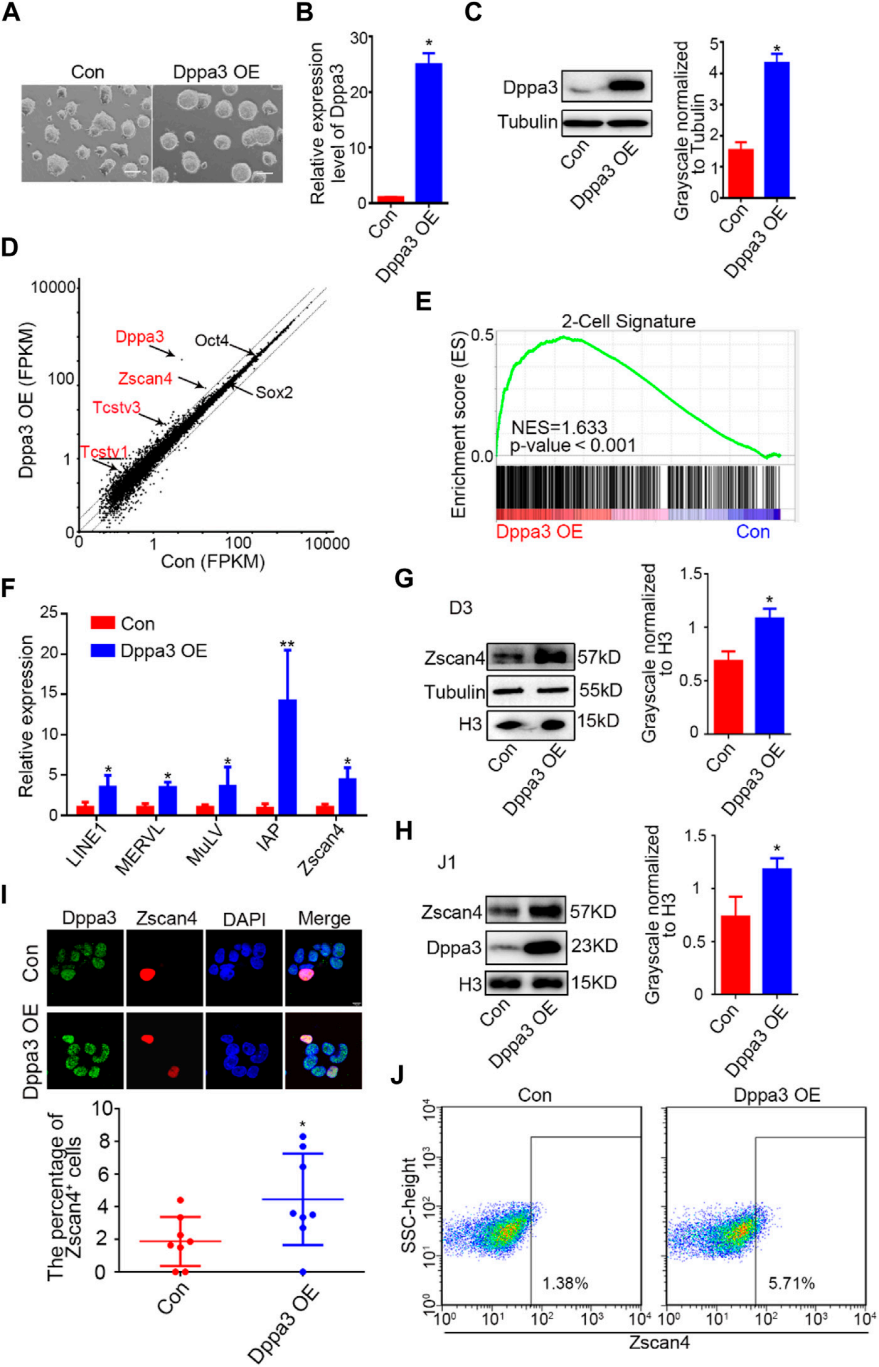
Overexpression of *Dppa3* activates a 2C-like gene expression. **(A)** Morphology of ESCs with an ectopic expression of *Dppa3*. Scale bars represent 100 μm. **(B)** Relative expression level of *Dppa3* in *Dppa3* OE and control (Con) ESCs. **(C)** Western blot analysis of Dppa3 protein level in *Dppa3* OE and Con ESCs. Right: Quantification of relative Dppa3 protein levels normalized to Tubulin by ImageJ software (*n* = 3; *p < 0.05). **(D)** Scatter plot showing differentially expressed genes following the ectopic expression of *Dppa3* in ESCs. Representative 2C genes that are upregulated in *Dppa3* OE ESCs are marked in red. Two-tailed Student’s *t*-tests were used to derive the *p* values. **(E)** GSEA indicating that upregulated genes in *Dppa3* OE ESCs were highly enriched in the two-cell embryo gene set. **(F)** qRT-PCR analysis of selected 2C-specific genes and retroviral elements expression in transient *Dppa3* OE ESCs. Data are presented as mean ± SEM (n = 3, *p < 0.05; **p < 0.01). **(G,H)** Western blot analysis of Zscan4 protein level in stable *Dppa3* overexpressed D3 **(G)** and J1 **(H)** ES cell lines. Right: Quantification of relative Zscan4 protein levels normalized to H3 by ImageJ software (*n* = 3; *p < 0.05). **(I)** *Dppa3* overexpression increases Zscan4 positive cells by immunofluorescence staining of Zscan4 (red) and Dppa3 (green) in stable *Dppa3* overexpressed D3 ESCs. Scale bar represents 10 μm. Below: Quantification of Zscan4 positive cells in *Dppa3* OE and control (Con) ESCs (n = 4; *p < 0.05; each dot corresponds to one replicate experiment, in which 200 cells were counted). **(J)** FACS profiles of Zscan4^+^ ESCs population in stable *Dppa3* OE ESCs.

Zygotes lacking maternal *Dppa3* activity fail to reach the blastocyst stage (Payer et al., 2003; Bortvin et al., 2004), demonstrating that Dppa3 plays a role during the cleavage stages of pre-implantation development. However, the contribution of Dppa3 to 2CLCs reprogramming in ESCs has not been studied in detail. We hypothesized that Dppa3 may regulate a 2C-like state. To address this hypothesis, we first generated ESCs with exogenous *Dppa3* overexpression (*Dppa3* OE ESCs). Morphologically, *Dppa3* OE ESCs showed compacted cell colonies (Figure 1A). Increased expression levels of *Dppa3* in *Dppa3* OE ESCs were confirmed by qPCR and western blotting (Figures 1B, C).

Next, we performed the mRNA-seq analysis. We identified 476 upregulated genes and 174 downregulated genes, respectively, more than a 1.5-fold change in *Dppa3* OE ESCs (Supplementary Table S4). RNA-seq results showed that transcripts of 2C-specific genes were significantly elevated in *Dppa3* OE ESCs, which were normally repressed by DNA methylation such as *Zscan4*, *Tcstv1*, and *Tcstv3* (Figure 1D). A gene set enrichment analysis for the stage specific genes also revealed 2C-specific genes were enriched in *Dppa3* OE ESCs (Figure 1E). The population of upregulated genes were also enriched with genes associated with germ cell development, such as spermatid development, spermatogenesis, and meiotic cell cycle in gene ontology (GO) terms (Supplementary Figure S1). We performed a detailed examination of the expression of 2C-like genes and endogenous transposable element (TE) in *Dppa3* OE ESCs, and found that *MERVL*, *LINE1,* and *IAP* were significantly activated in *Dppa3* OE ESCs (Figure 1F). MERVL-LTR transcriptional network activation occurs not only in two-cell embryos *in vivo* but also *in vitro* in a subset of mouse ESCs and during iPSC reprogramming (Eckersley-Maslin et al., 2016). Zscan4, expressed transiently in sporadic ESCs (1–5%) at any given time, marks transient 2C-state cells (Zalzman et al., 2010; Rodriguez-Terrones et al., 2018). The most significant difference in the transcription factor expression between the totipotent 2CLCs and the pluripotent ESCs was the Zscan4 expression (Falco et al., 2007). So, we next investigated the expression pattern of Zscan4 in *Dppa3* OE ESCs. Western blot results indicated that *Dppa3* OE promoted the expression of Zscan4 (Figure 1G). The elevation protein level of Zscan4 in *Dppa3* OE ESCs was also verified in another ESCs’ line (J1ES cells) (Figure 1H). We next examined the percentage of Zscan4-positive (Zscan4^+^) cells in *Dppa3* OE ESCs. An increased percentage of Zscan4^+^ ESCs in *Dppa3* OE ESCs was detected by immunofluorescence (IF) and cytometry analysis (Figures 1I,J; Supplementary Figure S2). Moreover, it can be seen from the immunofluorescence results that Zscan4^+^ ESCs also have a higher intensity of Dppa3 signal (Figure 1I). These findings suggested that the ectopic expression of *Dppa3* activated the 2C-specific genes and enhanced the percentage of 2CLCs in ESCs.

### Dppa3 Is Required for 2CLCs Conversion in ESCs

**FIGURE 2:**
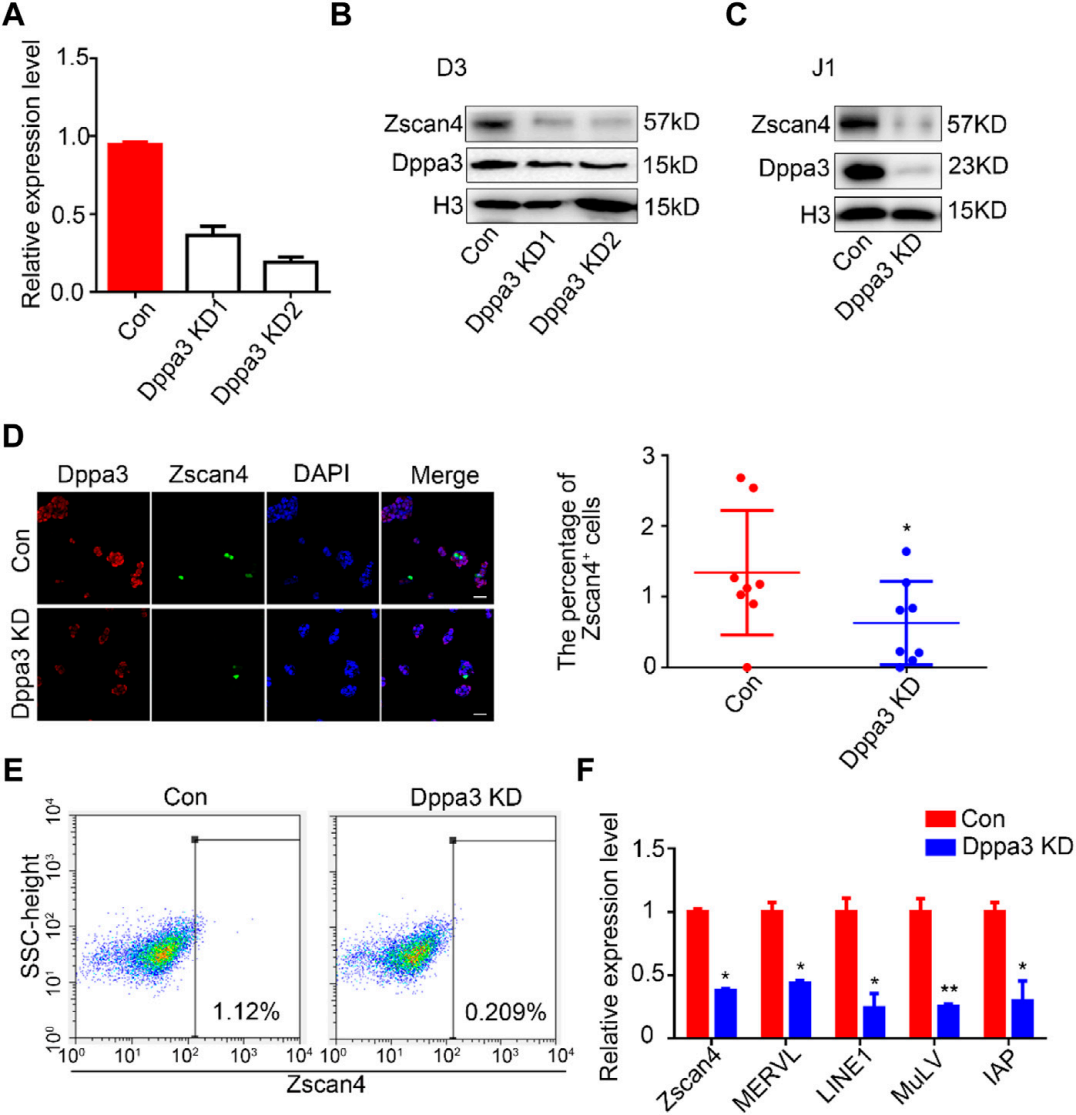
Knockdown of *Dppa3* impair the 2CLCs conversion in ESCs. **(A)** Confirmation of the expression level of *Dppa3* in transient *Dppa3* KD and control ESCs by quantitative PCR (n = 2, *p < 0.05). **(B,C)** The transient knockdown of *Dppa3* decreases the Zscan4 protein level analyzed by western blot in D3 ESCs **(B)** and J1 ESCs **(C)**. **(D)** The transient *Dppa3* KD reduces the Zscan4 positive cells by immunofluorescence staining of Zscan4 (green) and Dppa3 (red) in ESCs. Scale bar represents 100 μm. Right: The number of Zscan4 positive cells was counted (*n* = 4; *p < 0.05; each dot corresponds to one replicate experiment, in which 200 cells were counted). **(E)** FACS profiles of Zscan4^+^ ESCs in transient *Dppa3* KD ESCs. **(F)** qRT-PCR analysis of selected 2C-specific genes and retroviral elements’ expression in transient *Dppa3* KD ESCs. Data are presented as mean ± SEM (n = 3, *p < 0.05; **p < 0.01).

To further evaluate the effect of Dppa3 in promoting the 2CLCs conversion, we generated *Dppa3* knockdown (*Dppa3* KD) ESCs by RNA interference (RNAi) and assessed the capacity for 2C-like state conversion. The mRNA and protein levels of Dppa3 in *Dppa3* KD ESCs were effectively reduced to about 30% of that in the control ESCs (Figures 2A, B). Compared with *Dppa3* KD1, the knockdown efficiency of *Dppa3* KD2 was more significant, so *Dppa3* KD2 ESCs were used for further analysis. Zscan4 protein levels were significantly decreased in *Dppa3* KD ESCs, compared to control ESCs, by using the western blot analysis (Figure 2B). It was also confirmed in another embryonic stem cell line, J1 ESCs, that the Dppa3 knockdown resulted in a decrease in Zscan4 protein levels (Figure 2C). Using immunofluorescence, we observed a pronounced reduction in the population of Zscan4^+^ cells in *Dppa3* KD ESCs (Figure 2D). Flow cytometry also showed a significant reduction of Zscan4^+^ cells in *Dppa3* KD ESCs (0.209%) compared with that in control ESCs (1.12%) (Figure 2E). We also examined the expression of 2C-related genes and endogenous transposable element (TE) in *Dppa3* KD ESCs, and found that *MERVL*, *LINE1,* and *IAP* were significantly reduced in *Dppa3* KD ESCs (Figure 2F). Taken together, combined overexpression of the *Dppa3* increased Zscan4^+^ cells, and we conclude that Dppa3 is a driver of the 2C-like state.

### *Dppa3* Overexpression Leads to DNA Hypomethylation in ESCs

**FIGURE 3:**
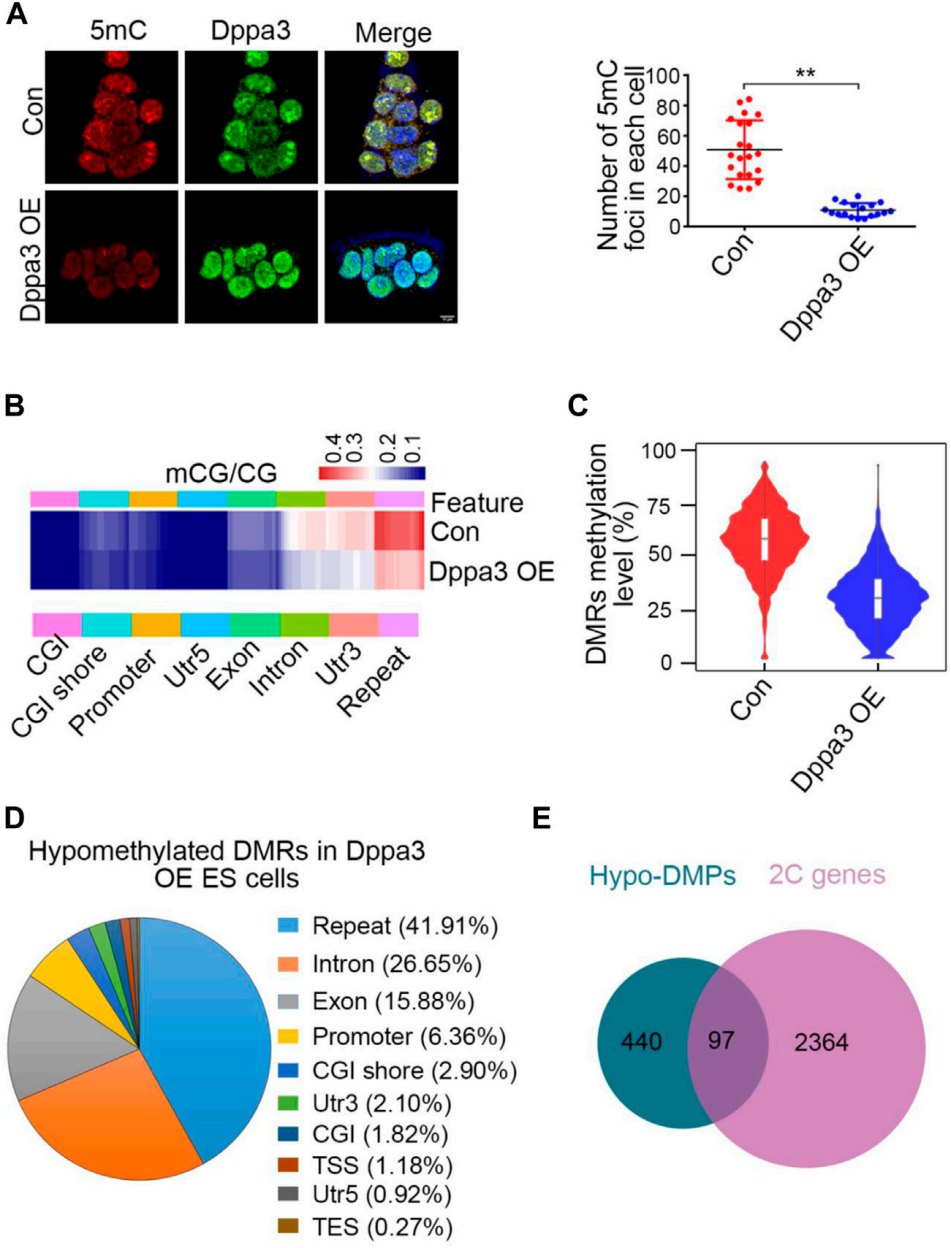
*Dppa3* overexpression leads to DNA demethylation in ESCs. **(A)** Immunofluorescence staining for 5mC (red) and Dppa3 (green) in *Dppa3* OE and Con ESCs. Right panel: quantification of 5mC foci in *Dppa3* OE and Con ESCs. Scale bar represents 10 μm. Data are presented as mean ± SEM (n = 3, ^**^ *p* < 0.01). **(B)** Hierarchical clustering of differentially methylated sites between *Dppa3* OE and control ESCs. Heat map displays methylation levels for whole genomes as measured by Reduced Representation Bisulfite Sequencing separated into groups by genomic features. Each row indicates a CpG site and the color scale represents the methylation level. CGI, CpG island; TSS, transcription start site; TES, terminate sequence. **(C)** DMRs methylation levels as measured by RRBS in *Dppa3* OE and control ESCs. Horizontal bars represent the median values. Inside is the boxplot. Violin flanking represents the number of genes at this methylation level. **(D)** The genomic distribution of hypomethylated DMRs in *Dppa3* OE ESCs. **(E)** Overlap between hypo-DMR-associated promoter genes (DMPs) and 2C-like genes.

DNA methylation globally loss in 2C-like cells (Dan et al., 2017). The molecular functions of Dppa3 on DNA methylation are demonstrated in zygotes, oocytes, and ESCs (Nakamura et al., 2012; Li et al., 2018; Mulholland et al., 2020), but little is known in 2CLCs conversion. To explore the underlying mechanisms of how 2C-like genes are activated in *Dppa3* OE ESCs, we next analyzed the DNA methylation pattern in *Dppa3* OE and control ESCs using immunofluorescence (IF) and reduced representation bisulfite sequencing (RRBS), respectively. While analyzing DNA methylation in ESCs with IF using a 5-methylcytosine (5mC) antibody, we noticed that DNA methylation levels were markedly lower in *Dppa3* OE ESCs compared to that in control ESCs (Figure 3A). The number of 5mC foci in each cell was also quantified, which was significantly reduced in *Dppa3* OE ESCs less than half of that in the control ESCs (Con), suggesting that the ectopic expression of *Dppa3* reduced the level of DNA methylation in ESCs (Figure 3A).

To unravel DNA methylation changes, we next performed RRBS of control and *Dppa3* OE ESCs to identify regions that are regulated by Dppa3. The RRBS analysis also revealed that the mCG levels were significantly reduced in *Dppa3* OE ESCs, mainly distributing at repeats, intron, and 3′UTR (Figure 3B). CpG island (CGI) showed the lowest levels of methylation difference between the *Dppa3* OE ESCs and the control ESCs, consistent with the fact that they are constitutively unmethylated in embryonic stem cells (Deaton and Bird, 2011). We specifically compared DMR (differentially methylated regions) methylation in *Dppa3* OE ESCs with that in the control ESCs. Approximate 60% of DMRs were methylated in control ESCs, and a lower proportion (30%) of methylated DMRs was found in the *Dppa3* OE ESCs (Figure 3C). DMRs were broadly distributed on all 19 autosomes (data not shown). The DMRs analysis identified 6,920 (98.2%) hypomethylated regions, whilst only 129 (1.8%) hypermethylated regions in *Dppa3* OE ESCs (Supplementary Tables S5, 6). We therefore focused on the hypomethylated DMRs (hypo–DMRs). The majority hypo–DMRs overlapped with repeat regions (∼2,900 regions, 41.91%). Only 6.36% of the regions mapped to the promoter (Figure 3D). The decrease in DNA methylation was particularly pronounced at LINE-1 (L1) elements. This widespread DNA hypomethylation was reminiscent for the global decrease in DNA methylation accompanying the 2CLCs reprogramming (Eckersley-Maslin et al., 2016). Next, we performed the gene ontology (GO) analysis using genes with differentially methylated promoters (DMPs). Among the hypomethylated DMPs (440), 97 genes (22%) were 2C-like genes (Figure 3E). These data strongly suggest that Dppa3 regulates DNA hypomethylation in ESCs, especially for 2C-like genes and retroviral elements.

### Ectopic Expression of *Dppa3* Increases DNA Damage Response in ESCs

**FIGURE 4:**
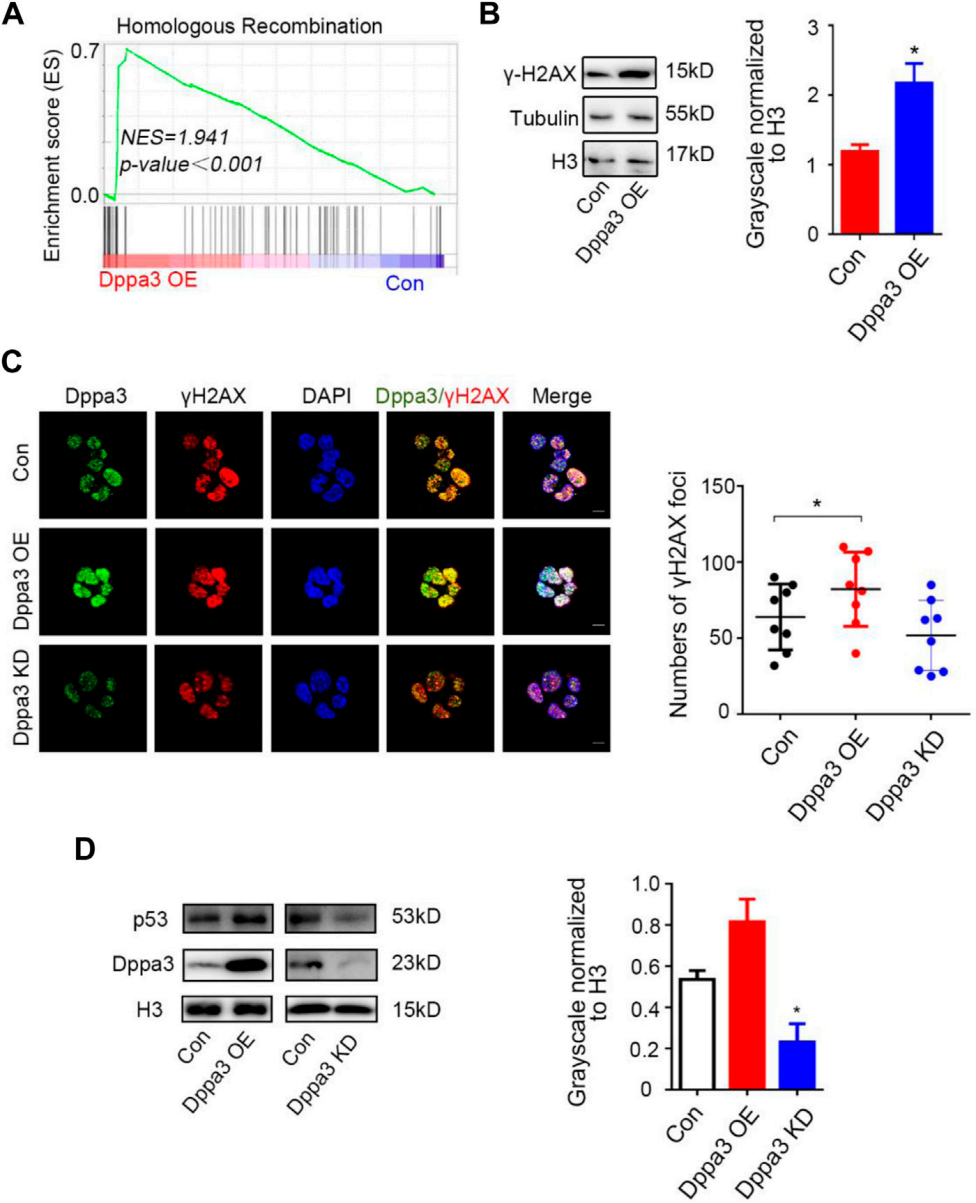
*Dppa3* induces DNA damage response in ESCs. **(A)** Gene set enrichment analysis (GSEA) indicating that upregulated genes in *Dppa3* OE ESCs are highly enriched in DNA recombination and repair pathway. **(B)** Western blot analysis of the level of γH2AX in *Dppa3* OE and control ESCs. Right: Quantification of relative γH2AX protein levels normalized to H3 by ImageJ software (*n* = 3; *p < 0.05). **(C)** Immunofluorescence analysis of γH2AX by staining for Dppa3 (green), γH2AX (red), and DAPI (blue) in *Dppa3* OE, *Dppa3* KD, and control ESCs. Scale bar = 10 μm. Right panel: Statistics of the number of γH2AX foci in each cell. The data are presented as mean ± SEM (*n* = 4; *p < 0.05; each dot corresponds to the numbers of γH2AX foci in each cell in one replicate experiment). **(D)** Western blot analysis of p53 in *Dppa3* OE, *Dppa3* KD, and control ESCs. Right: Quantification of relative p53 protein levels normalized to H3 by ImageJ software (*n* = 3; *p < 0.05).

Remarkably, by the GSEA analysis, DNA damage and recombination pathways were also enriched in *Dppa3* overexpression ESCs (Figure 4A). Recently, it has been reported that DNA damage is required for 2C-like cells’ induction in ESCs (Grow et al., 2021). p53 has been shown to be necessary for DNA damage-mediated Dux induction and the emergence of 2CLCs (Atashpaz et al., 2020; Grow et al., 2021). There are many sources of endogenous DNA damage in early embryos (Ziegler-Birling et al., 2009; Srinivasan et al., 2020), among which p53 has been found to be activated shortly after fertilization. p53 plays an important role in Dux activation and Dux targeted expression during ZGA, and the loss of p53 can lead to the reduction of endogenous fluctuating 2CLCs (Storm et al., 2014; Hendrickson et al., 2017; Grow et al., 2021). Phosphorylated H2AX (γH2AX) is also commonly used as a DNA damage response marker. To assess whether Dppa3 is responsible to DNA damage response, we performed immunofluorescence and western blot analysis of γH2AX and p53 in *Dppa3* OE and *Dppa3* KD ESCs. The immunofluorescence intensity and protein levels of γH2AX and p53 were both significantly increased following by *Dppa3* overexpression and decreased by *Dppa3* knockdown in ESCs (Figures 4B–D). Immunofluorescence results also indicated that Dppa3 closely co-localized with γH2AX (Figure 4C), demonstrating that Dppa3 promotes DNA damage response in ESCs.

### Dppa3 Facilitates the Chromatin Conversion to Resembling That of Two-Cell Embryos

**FIGURE 5:**
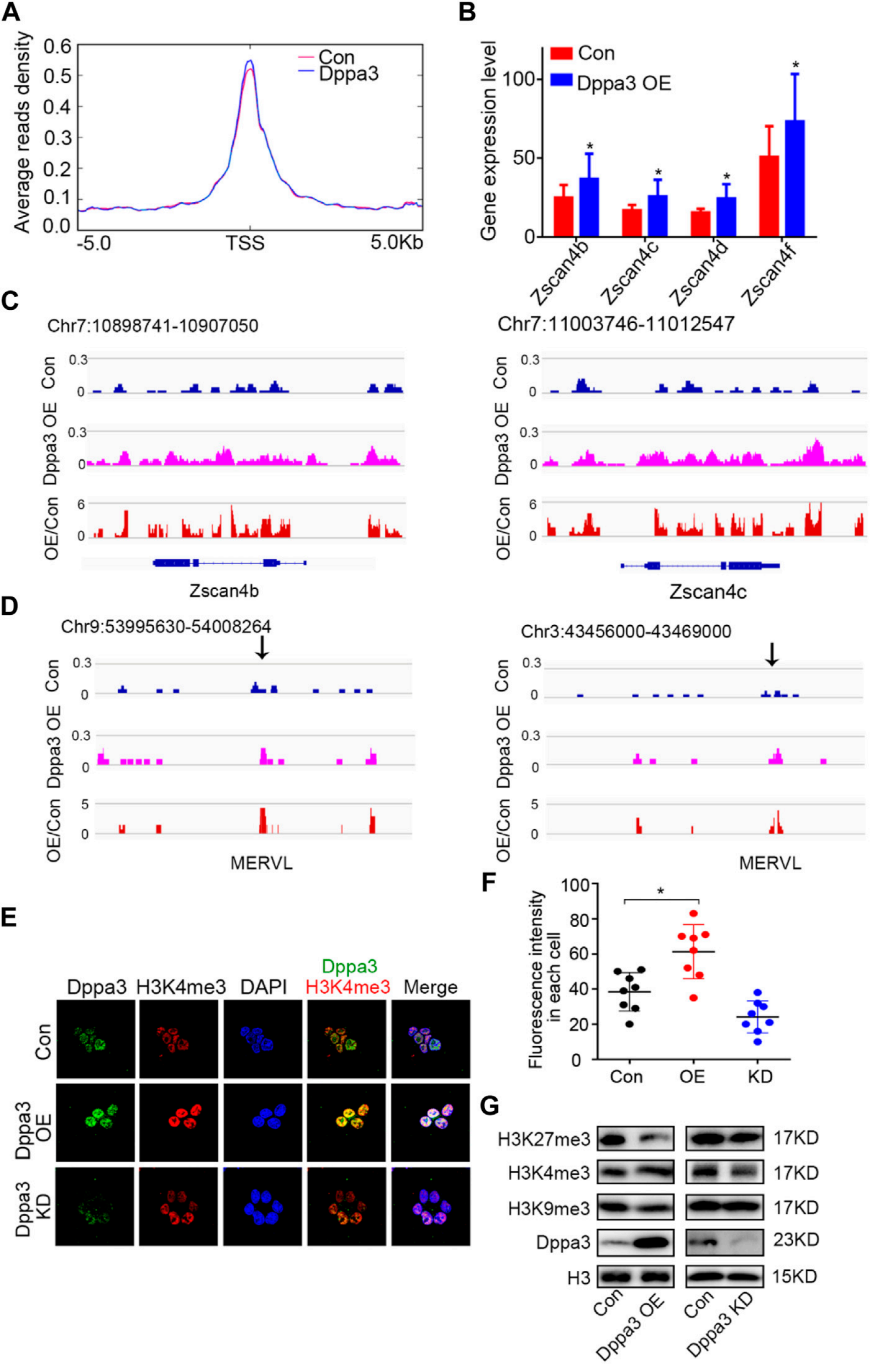
*Dppa3* facilitates the chromatin conversion to resembling that of two-cell embryos. **(A)** Average density of ATAC-Seq reads around TSSs (±5 kb) for the 2C related gene set. The red line represents the signal distribution of ATAC in 2C-specific genes set in Con group, the blue line represents the signal distribution of ATAC in 2C-specific genes set in the *Dppa3* OE group. **(B)** Expression of *Zscan4b*, *Zscan4c*, *Zscan4d*, and *Zscan4f* in *Dppa3* OE ESCs compared to those in Con ESCs. **(C,D)** Genome browser view of the distribution of ATAC-Seq signals at the 2C-related genes in *Dppa3* OE and Con ESCs. Shown are *Zscan4* (at Chr7) **(C)** and *MERVL* (at Chr3 and Chr9) **(D)**. **(E)** Immunofluorescence of H3K4me3 (red) and Dppa3 (green) in *Dppa3* OE, *Dppa3* KD, and control ESCs. Scale bar = 10 μm. **(F)** Quantification of numbers of H3K4me3 foci in *Dppa3* OE and Con ES cells (n = 4; *p < 0.05; each dot represents the fluorescence intensity in each cell in one replicate experiment). **(G)** Western blot analysis of histone modifications in *Dppa3* OE, *Dppa3* KD, and control ESCs.

DNA demethylation always couples with histone modification and chromatin configuration. Next, we profiled the chromatin accessibility of *Dppa3* OE ESCs by assay for transposase-accessible chromatin sequencing (ATAC-Seq) (Li et al., 2019; Yan et al., 2020). After peaks in each condition were called, regions of different ATAC sensitivities were identified. We identified 3,117 regions that gained an ATAC signal (ATAC gained) in *Dppa3* OE ESCs compared to that in Con ESCs. Furthermore, we found that *Dppa3* OE cells exhibited more extensive and specific opening of chromatin specifically at the 2CLCs-related gene set (Figure 5A). The *Zscan4* cluster has nine paralogous genes (including pseudogenes) located at heterochromatin (sub-telomere region). Three of them, *Zscan4c*, *Zscan4d*, and *Zscan4f*, encode full-length ORFs with 506 amino acids. Through the analysis of RNA-seq, we found that *Dppa3* overexpression elevated the transcription level of *Zscan4* (Figure 5B; Figure 1D). *Zscan4* was activated after *Dppa3* overexpression in ESCs and has been identified by prior studies as the marker of the 2C-like states (Figure 1). Our ATAC-Seq results also indicated that *Zscan4* elements are ATAC signal-enriched loci in *Dppa3* OE ESCs (Figure 5C). Like *Zscan4*, *MERVL* elements*,* which are located near the heterochromatin regions, are also ATAC signal-enriched loci in *Dppa3* OE ESCs, further suggesting that *Dppa3* facilitate the epigenomic remodeling especially for the heterochromatin (Figure 5D). *Dppa3*-induced 2C-like cells exhibited an extensive and specific opening of chromatin at *MERVL* and *Zscan4* elements, which are specifically expressed in 2C stage embryos to regulate 2CLCs conversion (Hendrickson et al., 2017).

**FIGURE 6:**
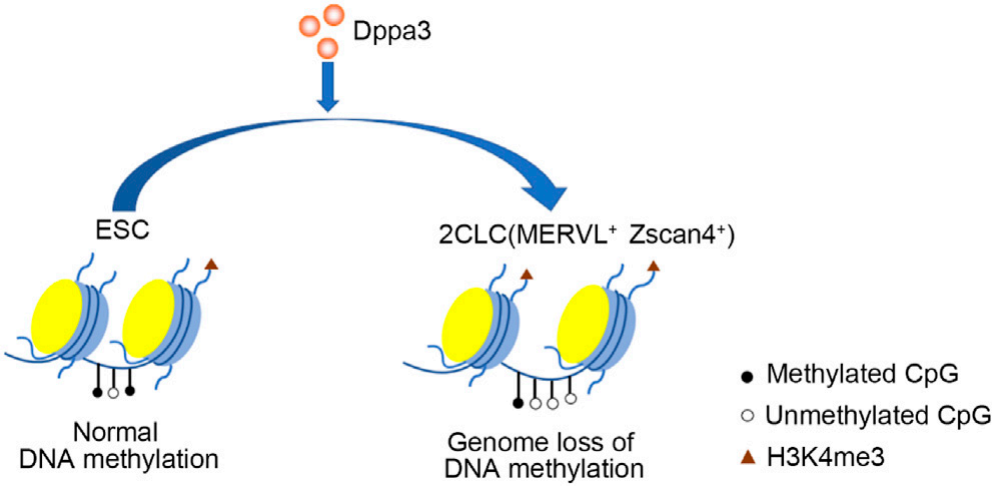
A model illustrating Dppa3-mediated DNA demethylation and 2C-like genes activation in ESCs. Dppa3 promotes chromatin remodeling, DNA hypomethylation, activates 2C-specific genes and facilitates 2CLCs conversion.

In previous observations, non-methylated CpG chromatin was usually enriched with H3K4me3. In the two-cell embryonic stage, the number of H3K4me3-marked promoters increased dramatically, and many promoters retained this modification in the following stages till the blastocyst stage. So, we next investigated the histone modification in *Dppa3* OE ESCs. Initially, we examined the H3K4me3 by immunofluorescence, which revealed that the global levels of H3K4me3 were increased in *Dppa3* OE ESCs (Figures 5E,F). Consistent with this, *Dppa3* knockdown reduced the H3K4me3 modification (Figures 5E,F). Furthermore, Dppa3 had a negative regulation on heterochromatin markers, such as H3K9me3 and H3K27me3 (Figure 5G), further indicating that Dppa3 promotes epigenetic remodeling in ESCs. Together, these data demonstrate that Dppa3 facilitates DNA hypomethylation in ESCs and induces ESCs acquiring chromatin accessibility at *Zscan4* and *MERVL* elements promoting 2C-like genes expression and 2CLCs conversion (Figure 6) (Eckersley-Maslin et al., 2016; Zhang et al., 2021).

## Discussion

The 2C-like state of ESCs recapitulates key aspects of the two-cell stage mouse embryo both phenotypically and molecularly (Eckersley-Maslin et al., 2018), which provides a cellular model to investigate the progress of ZGA. A main finding of this study is that Dppa3 promotes 2CLCs reprogramming in ESCs. Dppa3 is a maternal inherit factor, lacking of which in oocytes, results in development defects before implantation, suggesting a key role of Dppa3 in early embryonic development (Payer et al., 2003; Bortvin et al., 2004; Nakamura et al., 2007). However, the molecular functions and mechanisms of Dppa3 in the regulation of 2CLCs conversion are not well demonstrated. To address this question, we generated and characterized *Dppa3* OE and KD ESCs, and found that *Dppa3* promotes the 2C-like gene expression. Dppa3 is required for DNA demethylation of repeat regions and maintenance of chromatin configuration at 2C-like genes. Collectively, the results indicate that Dppa3 promote locus-specific demethylation and facilitate 2C-state conversion.

In mice, two-cell embryos are really totipotent cells that can develop into embryos and all the supporting extra-embryonic tissues (Rossant and Tam, 2017), but this kind of totipotent state is a transient state, which is difficult to carry out experimental research (Macfarlan et al., 2012). 2CLCs with the characteristics of two-cell embryonic cells and multidirectional differentiation potential found in ESCs culture are an excellent model for investigating ZGA. Previous studies of 2CLCs revealed some factors that mediate the activation of ZGA. As a development-related factor, Dppa3 is heterogenetic expressed in ESCs. The expression of Dppa3 increased in the naive state ESCs (Sang et al., 2019). Ogawa *et al.* found that when reprogramming via somatic nuclear transfer to produce cloned embryos, a large number of embryos were blocked in the two-cell embryonic stage, and these cells were characterized by a low *Dppa3* expression (Ogawa et al., 2015). Entry into the two-cell embryo is accompanied by DNA demethylation (Ishiuchi et al., 2015; Eckersley-Maslin et al., 2016). 2CLCs also have a distinct transcriptome profile, more accessible chromatin, reduced DNA methylation, and fewer repressive marks (Eckersley-Maslin et al., 2016; Eckersley-Maslin et al., 2018; Rodriguez-Terrones et al., 2018; Genet and Torres-Padilla, 2020). The molecular functions of Dppa3 on DNA methylation are well demonstrated in zygotes and oocytes (Nakamura et al., 2012; Li et al., 2018). Dppa3 also drives global DNA demethylation in ESCs (Mulholland et al., 2020), but little is known in 2CLCs. Our methylome analysis of *Dppa3* OE ESCs revealed 6,920 hypo-DMRs, only 129 hyper-DMR were detected. Moreover, Dppa3-dependent hypo-DMRs are enriched at 2C-like genes. Activation of 2C-like genes in *Dppa3* OE ESCs might rather reflect a role of Dppa3 during the early two-cell stage in regulating DNA methylation. 2CLCs display higher levels of some histone modifications associated with transcriptional activation. In addition, 2CLCs are similar to embryonic cells of the two-cell stage with a highly loose chromatin structure (Macfarlan et al., 2012; Ishiuchi et al., 2015; Genet and Torres-Padilla, 2020). H3K4me3 is known to form atypical broad domains in mouse two-cell stage embryos, and is essential for ZGA progress (Dahl et al., 2016). Our ATAC-Seq analysis showed that 2C-specific genes have a more open chromatin configuration in *Dppa3* OE ESCs, suggesting that Dppa3 can induce the production of 2CLCs by promoting the chromatin opening of 2C-related genes.

Studies have shown that *Zscan4* and *MERVL* are the key characteristics of the two cell embryos (Macfarlan et al., 2012). Zscan4 can promote the repair of DNA damage and the correction of chromosomal abnormalities, and plays a crucial role in maintaining genome and chromosomal integrity before embryo implantation (Ko, 2016). However, during pre-implantation development, RNA and protein products of Zscan4 were not detected in oocytes or zygotes, but were abundant in late-stage two-cell embryos (Falco et al., 2007), suggesting its expression is regulated by some maternal factors. *Dppa3* is expressed in the oocyte at both mRNA and protein levels, indicating a role of Dppa3 during the two-cell stage development and ZGA. Using overexpression and knockdown of *Dppa3* in ESCs, we found that Dppa3 activates the 2C-like gene expression, including *Tcstv1/3*, *Zscan4,* and *MERVL*, indicating a positive regulation of *Dppa3* in 2CLCs conversion and ZGA.

## Conclusion

Collectively, the data presented here demonstrate another previously unknown role for Dppa3 in the early embryo contributing to the two-cell stage genes’ activation. Dppa3 activates ZGA-specific gene expression and promotes the 2CLCs conversion.

## Materials and Methods

### Cell Culture

Mouse ESCs (J1 or D3) were all cultured in high glycose DMEM (HyClone) supplemented with 15% fetal bovine serum (HyClone), 2 mM l-glutamine (Gibco), 5,000U/mL penicillin and streptomycin (Gibco), 0.1 mM NEAA (Gibco), 0.1 mM 2-mercaptoethanol (CAS No. 60-24-2, Sigma), and 1,000U/ml LIF (ESG1107, Millipore Crop) at 37 °C in 5% CO2 incubator.

### Generation of Dppa3 Overexpression ES Cells

Murine *Dppa3* CDS was cloned into expression vector pLch3.7 (CAG promoter). pLch3.7–*Dppa3* and empty vectors (served as control) were transfected into ESCs by Lipofectamine 2000 (Invitrogen) according to the manufacturer’s instructions. After 2 weeks selection (1.5 μg/ml puromycin), *Dppa3* overexpression (*Dppa3* OE) ESCs colonies were picked and cultured in the ESCs culture medium for stable *Dppa3* OE ESCs lines. ESCs were transfected with pLch3.7-*Dppa3* or empty vectors and then selected by puromycin (2 μg/ml) for 24 h, which were used as transient *Dppa3* overexpression ESCs.

### Generation of *Dppa3* KD ES Cells by RNA Interference

Control (Con) and shRNA sequences (Supplementary Table S3) against *Dppa3* mRNA were used for the *Dppa3* knockdown (*Dppa3* KD) experiment. The sequences were cloned into pSIREN-RetroQ vector (Clontech). The reconstructed pSIREN-RetroQ vectors were then transfected into ESCs using Lipofectamine 2000 (Invitrogen). The cells were then cultured by the non-penicillin or streptomycin culture medium for 2 days. ESCs were then selected by puromycin (1.5 μg/ml) for 24 h, and positive clones were next used in the experiment.

### Flow Cytometry Analysis

To analyze the endogenous Zscan4 expression profile (percentage of Zscan4^+^ cells and fluorescence intensity), ESCs were collected and washed with cold PBS, then fixed in cold 4% PFA, permeabilized in 0.1% Triton X-100 in blocking solution (4% BSA in PBS) for 30 min, washed three times, and left in the blocking solution for 1 h. ESCs were incubated 1 h at room temperature with the primary antibody against Zscan4 (1:1000, Millipore), washed three times, and incubated for 30 min with secondary antibodies, FITC goat anti-rabbit IgG (1:1000, Beyotime). Samples were washed three times with PBS and the FACS analysis was performed using a FACSAria Flow Cytometer (BD Biosciences).

### Immunofluorescence Staining

ESCs were fixed in 4% paraformaldehyde for 30 min at room temperature and then permeabilized with 0.1% Triton X-100 for 30 min. Then cells were blocked in 10% goat serum for 1 h at room temperature. Primary antibodies against Dppa3 (1:200; R&D systems, MAB2566), Zscan4 (1:1000, Millipore, AB4340), H3K4me3 (1:1000, Abcam, ab8580), γH2AX (1:1000, Cell Signaling Technology, 80312), and 5mC (1:200, Abcam, ab10805, the cells were treated with 2 M HCl for 30 min, then neutralized with Tris-HCl of pH 8.0) were diluted in 1% normal serum (v/v) and incubated overnight at 4 °C and detected with the respective secondary antibodies including Alexa Fluor 594 secondary antibody (1:500; Proteintech, SA00006-4), Alexa Fluor 647 secondary antibody (1:500; Abcam, ab150159), Alexa Fluor 555 secondary antibody (1:500; Beyotime, A0460) at room temperature for 1 h and labeled with DAPI (Sigma) to visualize the nuclei. Sample images were captured using laser scanning confocal microscopy.

### RNA Extraction and Real-Time PCR Analysis

Total RNA was prepared using TRIzol (Invitrogen) and samples were treated with DNase (DNA-free, Ambion) following the instructions. 2 μg RNA was reverse-transcribed using the first-strand cDNA Synthesis System (Roche) with oligonucleotides. Real-time PCR was performed using the TransStart Green qPCR SuperMix Kit (TransGen Biotech). All quantitative PCR (qPCR) reactions were performed in the Opticon® System (Bio-Rad). Primer sequences are listed in Supplementary Table S2. The expression level of genes was determined from the threshold cycle (CT), and the relative expression levels were normalized to the expression of *18S* ribosomal RNA, respectively, and calculated by the 2^−ΔΔCT^ method as described in the literature.

### Western Blot

ESCs were harvested and lysed in the Western lysis buffer (Beyotime). For Western blots, Samples were boiled at 95 °C for 10 min with 1×SDS sample buffer before loading onto 4–20% Tris-glycine gels (Bio-Rad). Resolved proteins were transferred to the polyvinylidene difluoride membrane (0.25 mm pore, Millipore). Membranes were then blocked for 1 h in 1% casein prepared in Tris-buffered saline and 0.1% Tween-20 (TBST) before blotting with respective primary antibodies diluted in TBST, overnight at 4 °C. Blots were washed three times with TBST and incubated with the secondary antibody in the same buffer for 1 h at room temperature. Post three TBST washes, the membranes were imaged on an automatic chemiluminescence image analysis system. Quantitation of signal and analysis was performed using the ImageJ software. The following primary antibodies were used: anti-Dppa3 (1:1000, Abcam, ab19878), anti-H3 (1:5000, Abcam, ab1791), anti-γH2AX (1:1000, Cell Signaling Technology, 80312), anti-Zscan4 (1:1000, Millipore, AB4340), and anti-Tubulin (1:10,000, Proteintech, 66031-1-Ig). The following secondary antibodies conjugated to horseradish peroxidase were used: goat polyclonal anti-rabbit IgG (Bio-Rad), and rabbit polyclonal anti-mouse IgG (Sigma, 1:5000).

### RNA-Seq

ESCs were harvested and total RNA was extracted using TRIzol (Invitrogen). Quality control of the extracted RNA, construction of an RNA-sequencing library and sequencing on BGISEQ-500 was performed at Shenzhen Genomics Institute (BGI). For the bioinformatics analysis, the clean reads were mapped to the Mus musculus mm10 reference genome using Bowtie2. Reads were assigned and counted to genes using the RSEM. Functional enrichment (GO annotation, KEGG, and GESA) of gene sets with different expression patterns was performed using cluster Profiler and GSEA (R version 4.1.0), respectively. Scatter plots were generated using Excel to graphically reveal genes that differ significantly between two samples. The RNA-seq data have been uploaded in GEO (GSE number: 197967).

### ATAC-Seq

After the ESCs were counted, they washed once with 50 ml of cold PBS. The nuclei from 100,000 ES cells were lysed in 50 μL of lysis buffer (10 mM Tris–HCl (pH 7.4), 10 mM NaCl, 3 mM MgCl2 and 0.1% NP-40). Immediately after lysis, the nuclei were spun at 500×g for 5 min to remove the supernatant. Nuclei were then incubated with Tn5 transposase and segmentation buffer (Illumina) at 37 °C for 30 min. After segmentation, the transposed DNA was purified with a Min Elute kit (Qiagen). Samples were then amplified by PCR. Post-PCR clean-up was performed by adding 1.2 volume of AMPure XP bead (Beekman). Library concentration was measured with Qubit. Library integrity was checked by the Agilent bioanalyzer, and 75 bp single-read sequencing was performed using an Illumina HiSeq 2,500 platform per standard operating procedures.

### Reduced Representation Bisulfite Sequencing

DNA concentration was measured using Qubit® DNA Assay Kit in Qubit® 2.0 Flurometer (Life Technologies). A total amount of 5.2 μg genomic DNA from each sample was digested using MspI (New England Biolabs, United States ), followed by end repair and adenylation. Cytosine-methylated barcodes were ligated to DNA following the manufacturer’s instructions. Fragments with the 40–220 bp size were selected by gel electrophoresis, purified, and bisulfite-treated using EZ DNA Methylation-Gold Kit (Zymo Research, United States ). The bisulfite converted DNA was then PCR amplified by KAPA HiFi HotStart Uracil + ReadyMix (2X). Library concentration was quantified by Qubit® 2.0 Flurometer (Life Technologies) and the insert size was assayed on an Agilent Bioanalyzer 2,100 system (Agilent Technologies, United States ). The library preparations were sequenced on an Illumina Hiseq (High Output Mode).

### Statistical Analysis

All presented results were obtained from at least three independent experiments for each condition. Data are expressed as mean ± SEM. Statistical analyses were performed by one- or two-way analysis of variance using GraphPad Prism software. Differences were considered statistically significant at *p* < 0.05.

## Data Availability

The original contributions presented in the study are included in the article/Supplementary Material, further inquiries can be directed to the corresponding authors.

## Abbreviations

ZGA, zygotic genome activation; MZT, maternal to zygotic transition; ESCs, embryonic stem cells; ICM, inner cell mass; 2CLCs, two-cell embryonic like cells; MERVL, mouse endogenous retroviruses (ERV)-L; Dppa3, developmental pluripotency-associated three; TE, transposable element; RNAi, RNA interference; IF, immunofluorescence; RRBS, reduced representation bisulfite sequencing; 5mC, 5-methylcytosine; DMR, differentially methylated regions; DMPs, differentially methylated promoters; γH2AX, phosphorylated H2AX; ATAC-Seq, transposase-accessible chromatin sequencing.
