# Supplementary material for: Maternal Factor Dppa3 Activates 2C-Like Genes and Depresses DNA Methylation in Mouse Embryonic Stem Cells
Source: Front Cell Dev Biol. 2022 Jun 3;10:882671. doi: 10.3389/fcell.2022.882671 (PMC9203971; doi:10.3389/fcell.2022.882671)
Supplement: Supplementary file 4 [file DataSheet1.docx]

Supporting Information ：

**Maternal factor Dppa3 activates 2C-like genes and depresses DNA methylation in mouse embryonic stem cells**

**Zhang *et al.***

**The Following Files are Included:**

**Supplementary Figures and Legends**

**Supplemental Figure 1**

**Supplemental Figure 2**

**Supplemental Table 1 *Dppa3* overexpression sequences**

**Supplemental Table 2 Primers used for quantitative RT-PCR**

**Supplemental Table 3 Interference Sequences**

**Supplemental Table 4 Differentially expressed genes in Dppa3 OE vs Con ES cells**

**Supplemental Table 5 Hypomethylated DMR in Dppa3 OE ES cells vs Con ES cells**

**Supplemental Table 6 Hypermethylated DMR in Dppa3 OE ES cells vs Con ES cells**

**Supplemental Figures:**


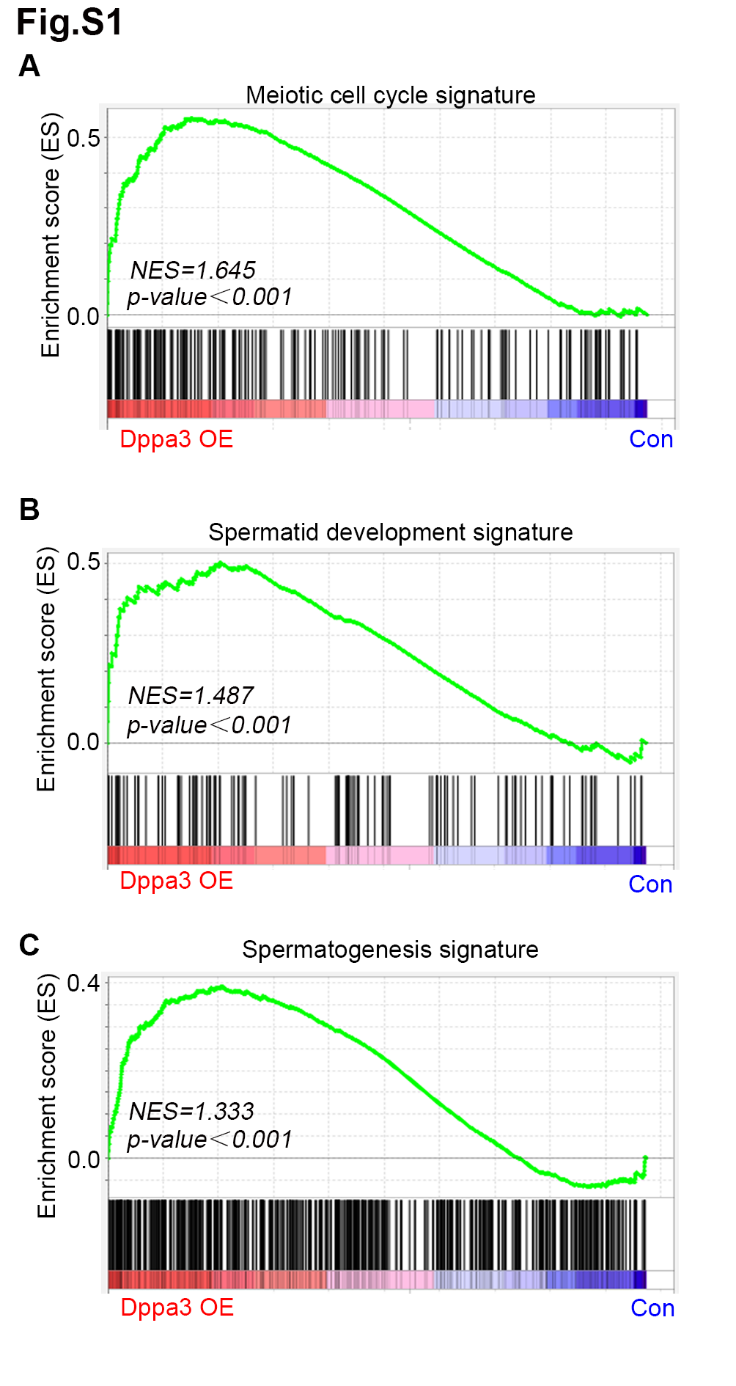


**Supplemental Figure 1:** GSEA indicating that upregulated genes in *Dppa3* OE ESCs were highly enriched in the meiotic cell cycle signature (**A**), spermatid development signature (**B**), and spermatogenesis signature gene set (**C**). Red, upregulated genes; blue, down regulated genes.


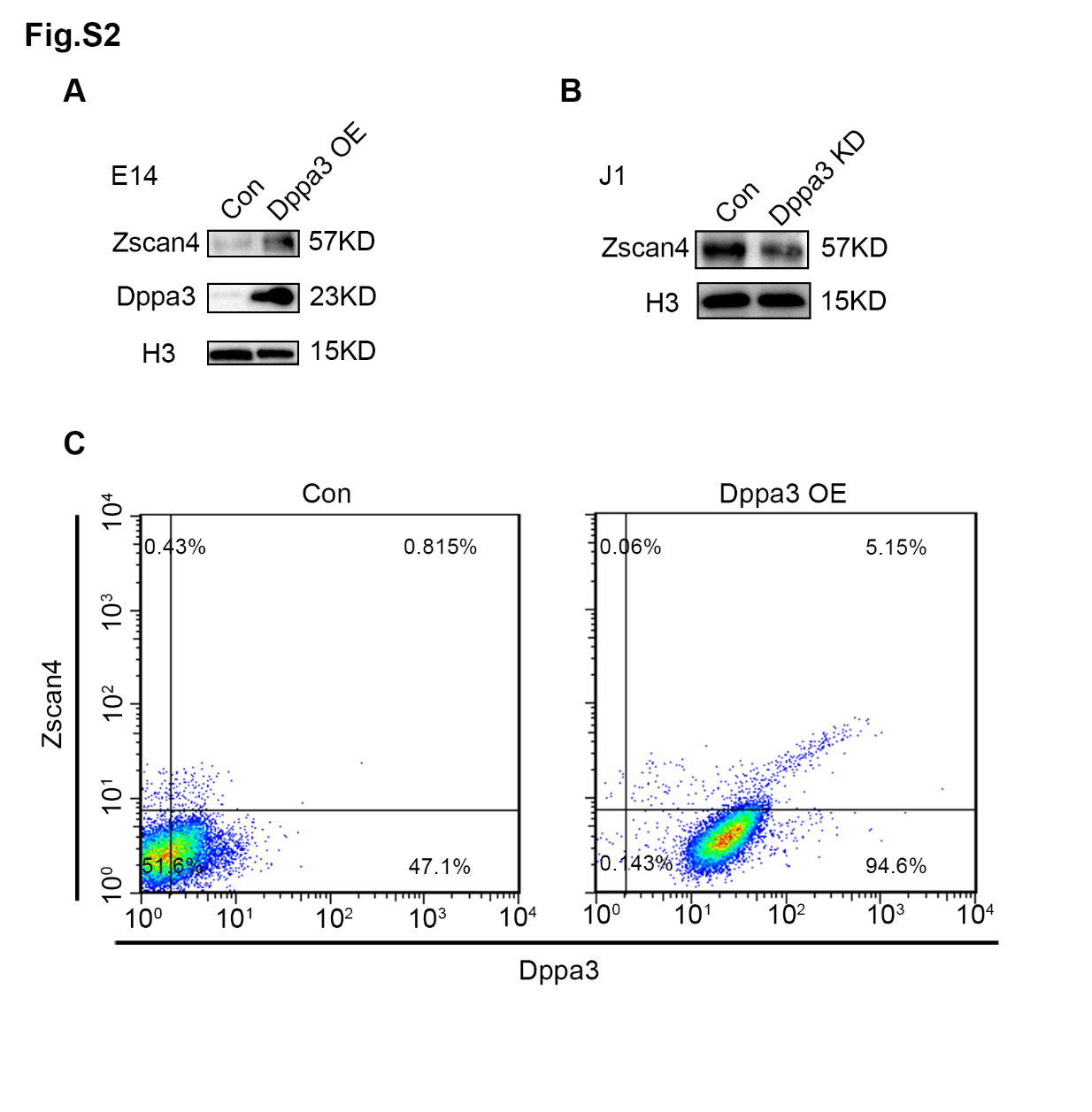


**Supplemental Figure 2:** **(A-B)** Western blot analysis of Zscan4 protein level in transient Dppa3-plasmid transfection in E14 **(A)** and J1 **(B)** ESCs. **(C)** FACS profiles of Zscan4^+^ and Dppa3^+^ ESCs population in transient Dppa3-plasmid transfection in D3 ESCs. ESCs were transfected with pCMV6--*Dppa3* and empty vectors (served as control). After 2 days, the cells were screened by Neomycin (1.2 mg/ml) for 24 hours and then used for FACS analysis.

**Supplemental Table 1 *Dppa3* overexpression sequences**

| Name | Sequence |
| --- | --- |
| *Dppa3* -F | CTAGCTAGCTGCTAATTGGGTCTTGG |
| *Dppa3* -R | ATACGATATCGAGGCTTCTAAACTTCACAT |

**Supplemental Table 2 Primers used for quantitative RT-PCR**

| Gene | Forward | Reverse |
| --- | --- | --- |
| *Dppa3* | CGGGACAGTGAGCCATTC | ACAGCCAGGGCAGCGTA |
| *18S* | GTAACCCGTTGAACCCCATT | CCATCCAATCGGTAGTAGCG |
| *MERVL* | ATCTCCTGGCACCTGGTATG | AGAAGAAGGCATTTGCCAGA |
| *MuLV* | GGCGCCCCGTACAAGATTTCATA | GATAACGGGCCTGCCTTCACCTC |
| *LINE1* | ATGGCGAAAGGCAAACGTAAG | ATTTTCGGTTGTGTTGGGGTG |
| *IAP-Gag* | AATCTCAGAACCGCTCCATGA | TTTCTTAAAATGCCCAGGCTTT |
| *Zscan4* | AGTGCTTGAAGCCTCCTGTC | GCCTTGTTGCAGATTGCTGT |

**Supplemental Table 3 Interference Sequences**

| Name | Sequences |
| --- | --- |
| *Dppa3* | GAAACTCCTCAGAAGAAAGAT |
| Scrambled | GCGTTCAATTAGCAGACCA |
